# Acute Necrotizing Encephalopathy: An Underrecognized Clinicoradiologic Disorder

**DOI:** 10.1155/2015/792578

**Published:** 2015-03-22

**Authors:** Xiujuan Wu, Wei Wu, Wei Pan, Limin Wu, Kangding Liu, Hong-Liang Zhang

**Affiliations:** ^1^Neuroscience Center, Department of Neurology, the First Hospital of Jilin University, Jilin University, Xinmin Street No. 71, Changchun 130021, China; ^2^Department of Neurovascular Surgery, the First Hospital of Jilin University, Jilin University, Xinmin Street No. 71, Changchun 130021, China; ^3^School of Public Health, Jilin University, Xinmin Street No. 1163, Changchun 130021, China; ^4^Neuroprotection Research Laboratory, Massachusetts General Hospital, Harvard Medical School, Charlestown, MA 02129, USA

## Abstract

Acute necrotizing encephalopathy (ANE) is a rare but distinctive type of acute encephalopathy with global distribution. Occurrence of ANE is usually preceded by a virus-associated febrile illness and ensued by rapid deterioration. However, the causal relationship between viral infections and ANE and the exact pathogenesis of ANE remain unclear; both environmental and host factors might be involved. Most cases of ANE are sporadic and nonrecurrent, namely, isolated or sporadic ANE; however, few cases are recurrent and with familial episodes. The recurrent and familial forms of ANE were found to be incompletely autosomal-dominant. Further the missense mutations in the gene encoding the nuclear pore protein Ran Binding Protein 2 (*RANBP2*) were identified. Although the clinical course and the prognosis of ANE are diverse, the hallmark of neuroradiologic manifestation of ANE is multifocal symmetric brain lesions which are demonstrated by computed tomography (CT) or magnetic resonance imaging (MRI). The treatment of ANE is still under investigation. We summarize the up-to-date knowledge on ANE, with emphasis on prompt diagnosis and better treatment of this rare but fatal disease.

## 1. Introduction

Acute necrotizing encephalopathy (ANE), first proposed by Mizuguchi et al. in 1995, is a rare but distinctive type of acute encephalopathy with global distribution [1]. It is generally considered to be a parainfectious disease that is triggered mainly by viral infections [1]. Most initially reported ANE cases were Japanese and Taiwanese children, leading to the suspicion that the disease was related to racial factors [2]. However, an increasing number of cases were later reported in Western countries [3–13] including adult ones [14, 15], suggesting a global distribution of ANE without racial predilection. ANE has yet been recognized primarily as a clinicoradiologic disorder with unknown etiology. We herein summarize the up-to-date knowledge of the etiology, pathogenesis, clinical manifestations, and radiological features, as well as current treatment and prognosis of ANE.

## 2. Etiology and Pathogenesis of ANE

At present, the etiology and the pathogenesis of ANE remain incompletely clear. Both environmental factors, which may contribute to the antecedent infections, and host factors such as individual susceptibility or alterations of genes might be involved [16]. Usually, ANE develops secondary to viral infections, including influenza A and influenza B, novel influenza A (H1N1), parainfluenza, varicella, human herpesvirus 6 and herpesvirus 7 (HHV-6 and HHV-7), enterovirus, novel reovirus train (MRV2Tou05), rotavirus, herpes simplex virus, rubella, coxsackie A9, and measles, among which the influenza virus and HHV-6 are most common [5, 9–13, 17–34]. Prodromal viral infections appear to play a critical role in the initiation of ANE. Despite the various antecedent infections, ANE is not considered to be an inflammatory encephalitis.

Although polymerase chain reaction (PCR) for some viruses was positive in cerebrospinal fluid (CSF) in some reports, there was no clue for encephalitis through autopsic pathological examinations, which showed notably minimal brain inflammation in relation to the marked parenchymal abnormality [3, 4, 20, 22, 25, 35]. Moreover, CSF pleocytosis is usually absent in patients with ANE. Additionally, no difference was noted with regard to clinical features and outcome except for the more frequent brain stem lesions in ANE secondary to influenza as compared with noninfluenza illnesses [36]. In this regard, development of ANE seems independent of the type of infectious agents.

Although the exact pathogenesis of ANE remains obscure, the most prevalent hypothesis is the hypercytokinemia, that is, “cytokine storm” [37–39]. Individuals suffering from ANE often have an exaggerated immune response to various viral infections by producing elevated proinflammatory cytokines resembling systemic inflammatory response syndrome (SIRS) [37, 38]. The “cytokine storm” results in systemic symptoms, such as liver dysfunction, acute renal failure, shock, and disseminated intravascular coagulation [37–39]. In the nervous system, it leads to brain injury through alteration of vessel wall permeability without vessel wall disruption [37, 38]. According to this hypothesis, ANE is an encephalopathy concomitant with systemic immune imbalance. Flow cytometric analysis on peripheral blood lymphocytes of patients with ANE revealed high proportion of CD56^+^ natural killer (NK) cells during the recovery phase [28]. These cells produced a high level of cytokines, suggesting that NK cells might be associated with the pathogenesis of ANE [28]. In addition, several lines of evidence showed that levels of cytokines were elevated in the serum and/or CSF in different virus-associated ANE including interleukin- (IL-) 6, tumor necrosis factor-alpha (TNF-*α*), IL-10, IL-15, IL-1*β*, soluble TNF receptor (sTNFR), and interferon-gamma (IFN-*γ*) [18, 20, 23, 26, 28, 38, 40–44]. IL-6 and TNF-*α* were critical among these cytokines, because the former was neurotoxic at high concentrations, whereas the latter could damage the endothelium of the central nervous system. Hypercytokinemia engenders proteolytic destruction of the blood-brain barrier (BBB) through the action of trypsin and the activation of matrix metalloprotease-9, which subsequently increases vascular permeability and causes brain edema, petechial hemorrhage, and necrosis [38, 44, 45]. Except for the above viral infections, ANE may also develop secondary to diphtheria, tetanus toxoid, and whole-cell pertussis (DPTw) vaccination [26]. The DPTw vaccination might result in increased production of cytokines and cause the alteration of vessel wall permeability, leading to local breakdown of BBB [38, 44–47].

ANE was initially believed to be with geographic predilection, whereas it was later found with global distribution [2, 3]. Most ANE cases are sporadic and nonrecurrent, namely, isolated ANE. However, few cases of recurrent and/or familial episodes have been reported suggestive of an inherited disposition [8, 48, 49]. In addition to environmental factors, host factors may contribute to the development of ANE as well. Genetic background, for example, human leukocyte antigen (HLA) DRB/HLA DQB genes might contribute to the pathogenesis of ANE [17]. Neilson and colleagues reported a case series with incompletely penetrant and autosomal-dominant acute encephalopathy identical to ANE; the dominance was estimated to be at 50% and recurrent episodes occurred in half of the affected individuals [48]. Further, by performing home linkage analysis they mapped the disease interval to human chromosome 2q12.1-2q13 sequencing for candidate genes (BCL2L11, ST6GalII, CHT1, and FLJ20019) that are involved in apoptosis, viral recognition, choline transport, and electron transport, whereas they failed to find any mutations [49]. Until 2009, their study had been extended to include 16 probands with familial ANE. The missense mutations in the gene encoding the nuclear pore protein Ran Binding Protein 2 (*RANBP2*) were found to be the susceptibility alleles for familial and recurrent cases of ANE, and they proposed naming this type of ANE as “ANE1” [50]. Since the relationship between ANE and the* RANBP2* gene was identified, there have been several reports on recurrent or familial cases, while the mechanism of this mutation leading to pathology remains unclear [20, 22, 51].* RANBP2* is located on the cytoplasmic surface of the nuclear pore, and its numerous functions throughout the cell cycle have been found, which include facilitation of the protein transportation and sumoylation of protein cargoes during interphase, intracellular trafficking, or energy maintenance in certain type of cells, including neurons, and contribution to the nuclear envelop breakdown as well as facilitation of sister chromatid resolution during mitosis [50]. The* RANBP2* mutations may affect intracellular trafficking of mitochondria or energy production and lipid peroxidation. It may also affect other processes including viral entry, antigen presentation, cytokine signaling, immune responses, and BBB maintenance [50]. However, the* RANBP2* missense mutations were not the sole susceptibility alleles for familial or recurrent ANE which accounted for 75% of the case series, because cases with recurrent ANE but without* RANBP2* missense mutations have also been reported [50–52]. Of note is that gray matter heterotopia and ANE may occur in patients with trichothiodystrophy (TTD), an autosomal-recessive disorder, arousing the hypothesis that ANE and TTD share a common genetic background [53].

Except for the above mentioned environmental and host factors, Shinohara et al. found that variations of the carnitine palmitoyl transferase II gene (*CPT II*) were associated with ANE [54]. Kumakura and colleagues found the thermolabile phenotype of carnitine palmitoyl transferase II in an ANE patient, and they speculated that it decreased enzymatic activities which reduced the utilization of mitochondrial fuel during high-grade fever [45]. Further, the impaired mitochondrial *β*-oxidation and the generation of adenosine triphosphate in the cerebral microvascular endothelial cells (ECs) engendered the increasing permeability of the vascular wall and the development of brain edema [45]. The increasing permeability of the vascular wall could be caused by genetic factors of susceptibility to energy failure in addition to pathophysiologic factors of hypercytokinemia.

Another interesting finding was the ephrin type B receptor 2 (EphB2), a novel cell-surface autoantigen which had critical functions to neuronal and ECs [55]. EphB2 was expressed by human brain microvascular ECs, which was found as a target of autoantibody from a patient with ANE complicated with systemic lupus erythematosus (SLE). Anti-EphB2 antibody, however, was not detectable in any SLE patients without ANE, indicative of its potentials as a biomarker of ANE. The possible mechanisms are as follows: (1) anti-EphB2 antibodies damage vascular ECs which results in breakdown and increased permeability of BBB; (2) when BBB is breached, anti-EphB2 antibodies exudate into brain tissue and bind neurons and neuroglia causing neuronal dysfunction and cell death [55]. However, the pathomechanism of anti-EphB2 antibodies in ANE remains largely unknown.

## 3. Clinical Manifestations of ANE

**Figure 1:**
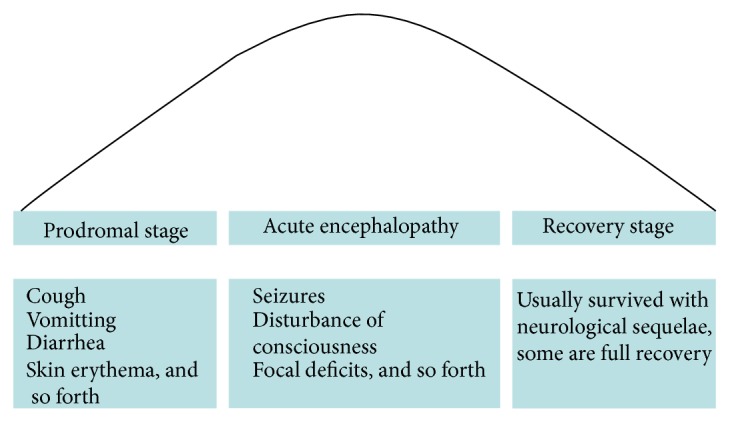
Clinical course of acute necrotizing encephalopathy (ANE). Survivors of ANE go through three phases during the clinical course including prodromal stage, period of acute encephalopathy, and recovery stage. In the prodromal stage, the common symptoms include cough, vomiting, diarrhea, skin erythra mainly due to various viral infections. Soon after, the dysfunction of the brain gradually appeared during the acute encephalopathy stage, for example, disturbance of consciousness, seizures, focal deficits. If survived, patients of ANE would go through the third phase, so-called recovery stage, and most patients left with different neurological sequelae while a few could recover completely.

Patients with ANE have neither specified symptoms nor typical neurological signs. Comparisons of the clinical features between Asian and non-Asian patients revealed homogeneity of the disease worldwide [12]. In addition to prodromal symptoms due to different viral infections, which include fever, signs of upper respiratory tract infections and gastroenteritis, and erythema, patients with ANE often have signs of SIRS like shock, multiple organ failure (MOF), and disseminated intravascular coagulation (DIC) [17, 33, 56]. With the development of ANE, brain dysfunctions may present as seizures, disturbance of consciousness, and focal neurological deficits [3, 17–26, 57]. Of note, however, is that none of the above manifestations are specific to ANE. Laboratory findings vary from case to case, while some could be used for differential diagnosis, such as abnormalities of liver function without hyperammonemia, hypoglycemia, or lactic acidosis. Moreover, the protein levels of CSF and platelet count could be a predictor of the prognosis of the disease [3, 17, 22, 31, 58]. The clinical course of ANE is fulminant and diverse, from a mild form with completely recovery or mild sequelae to a severe form with a high mortality [25, 31, 37, 59, 60]. Usually, survivors of ANE go through three phases during the clinical course including prodromal stage, period of acute encephalopathy, and recovery stage as shown in Figure 1 [5, 7, 9, 10, 18–20, 23, 29, 31, 35, 61–63]. Due to the decreased incidence of autopsies, the diagnosis of ANE was mainly based on characteristic neuroradiologic findings [3, 64, 65]. However, diagnosis can only be established pending the exclusion of other resembling diseases. Except for the genetic background, familial ANE is similar to isolated ANE in terms of clinical and radiologic features [50]. However, recurrences produce more severe functional impairments [8, 48–51, 61]. The diagnostic criteria and differential diagnoses of ANE are shown in Section 8.

## 4. Neuroradiologic Features of ANE and Its Clinical Significance

The hallmark of neuroradiologic manifestations of ANE is multifocal, symmetric brain lesions involving both the gray matter and the white matter that are demonstrated by computed tomography (CT) or magnetic resonance imaging (MRI), consistent with histopathologic findings via autopsy [2]. The topographic distributions are remarkably similar among patients with ANE, including thalami, brain stem, cerebral white matter, and cerebellum [2, 18–25, 64, 65]. Bilateral thalami are typically involved in all patients with ANE, serving as a distinctive feature of ANE [2, 58]. Spinal cord may occasionally be involved as well [20, 61, 66].

**Figure 2:**
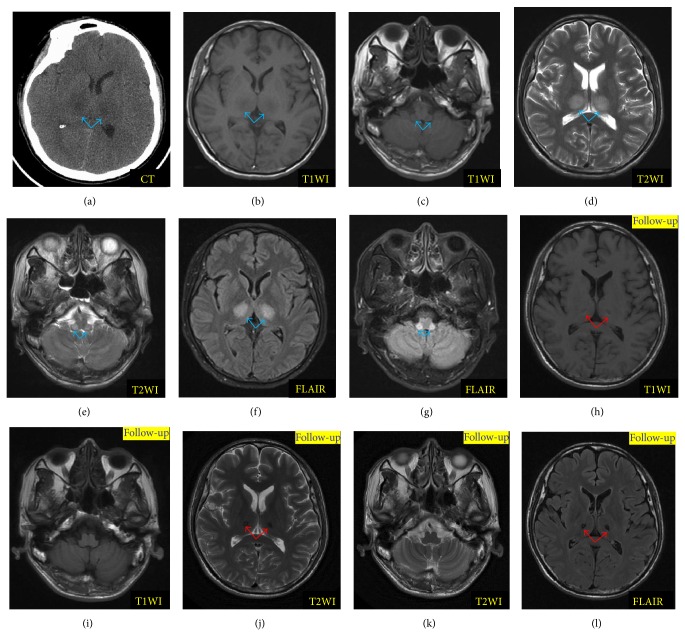
Dynamic changes of magnetic resonance imaging (MRI) of a patient with acute necrotizing encephalopathy (ANE). (a) was computerized tomography (CT) at onset; (b) and (c), (d) and (e), and (f) and (g) were, respectively, the T_1_-weighted image (T_1_WI), T_2_WI, and fluid attenuated inversion recovery (FLAIR) image at onset which showed lesions on bilateral thalamus and brain stem (blue arrow); (h) and (i), (g) and (k), and (l) were, respectively, the T_1_WI, T_2_WI, and FLAIR imaging of follow-up which revealed disappearance of the brain stem lesions and impressive regression of the thalamic lesions, just left hemosiderin deposition (red arrow).

**Figure 3:**
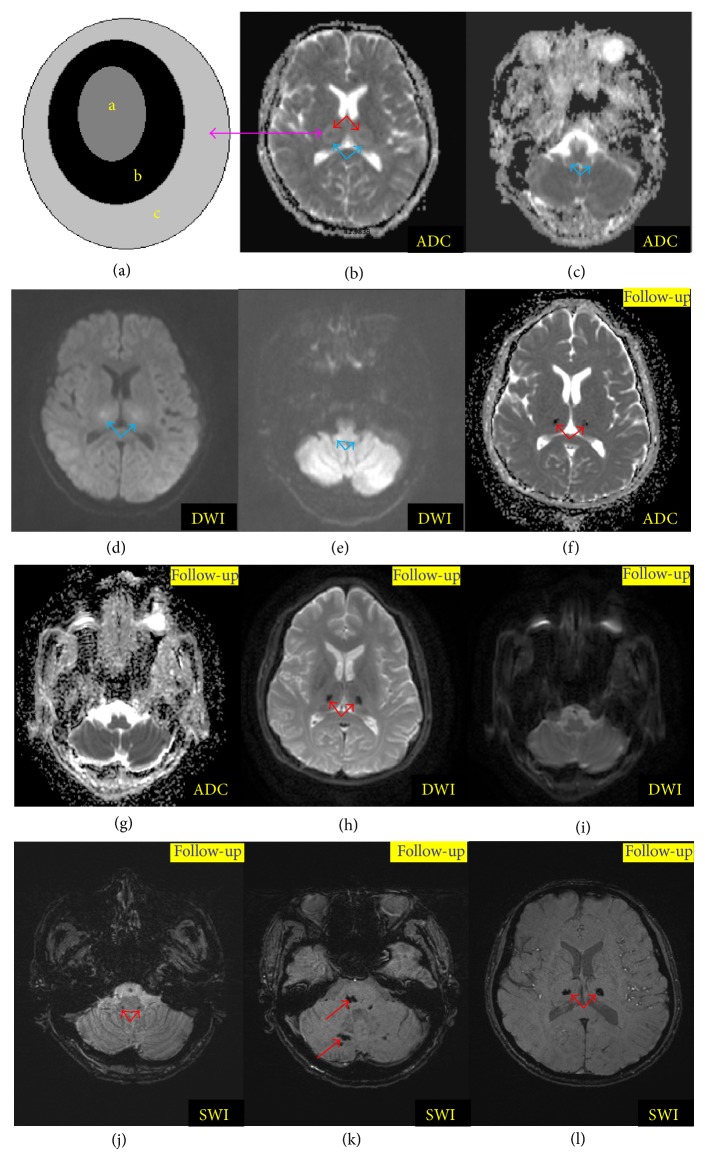
Diffusion MRI and susceptibility weighted imaging (SWI) findings of acute necrotizing encephalopathy (ANE). (a) was the schematic diagram of typical tricolor pattern corresponding to the thalamic lesions on (b) (a: center of thalamic lesions characterized by hemorrhage and necrosis; b: periphery of the central thalamic lesions characterized by cytotoxic edema; c: outside portions of the thalamic lesions suggesting vasogenic edema). (b) and (c), (d) and (e) were the apparent diffusion coefficient (ADC) and diffusion-weighted image (DWI), respectively, at onset which suggested the bilateral thalamus and brain stem lesions (blue arrow). (f) and (g), (h) and (i) were the ADC and DWI imaging of follow-up which revealed disappearance of the brain stem lesions and hemosiderin deposition on bilateral thalamus (red arrow). (j), (k), and (l) were the follow-up SWI images which showed hemosiderin deposition in the bilateral thalami and the cerebella (red arrow).

Neuroradiologic manifestations are characterized by dynamic changes during the clinical course corresponding to pathophysiological changes from edema to petechial hemorrhage and then to necrosis [1, 2]. Regression or recovery of the brain lesions is possible for survivors [1–4]. Herein we explain the dynamic imaging changes in the brain of ANE by exhibiting the neuroimages of our patient with ANE (Figures 2 and 3). Lesions in the brain are edematous and combined with mass effect at the onset of ANE. Hypodensities are frequently seen on CT (Figure 2(a)) and homogeneously prolonged T_1_ (Figures 2(b) and 2(c)) and T_2_ (Figures 2(d) and 2(e)) relaxation time (Figures 2(b) and 2(c)) of the brain lesions on MRI are found in most patients. Moreover, the feature of restricted water diffusibility on diffusion MR including diffusion-weighted imaging (DWI) and apparent diffusion coefficient (ADC) (Figures 3(b), 3(c), 3(d), and 3(e)) can be found in a majority of ANE patients [10, 11, 18–20, 22–26, 35–37, 45, 67]. Gradually, with the resolution of edema and mass effect, the feature of petechial hemorrhage and necrosis appears, and hypodense areas on CT become mottled because of the irregular hyperdense spots at the center which result from the extravasation of blood vessels or petechial hemorrhage [35, 59]. On the corresponding T_1_-weighted imaging (T_1_WI), increased signal intensities in the center surrounded by the decreased signals are detected, while T_2_-weighted imaging (T_2_WI) may reveal decreased signal intensities that are surrounded by increased or homogeneous increased signal intensities [5, 10, 26, 67]. Small petechial hemorrhage is usually obscure. The T_2_^*^-weighted gradient echo imaging or the susceptibility weighted imaging (SWI) is more sensitive in showing the petechial hemorrhage of ANE, both of which demonstrate low signal intensities [9, 67, 68].

The classical neuroimaging of ANE was “concentric/laminar structure” or “tricolor pattern” or target-like appearance [6, 22, 64, 65]. This typical manifestation is more obvious on ADC of MRI (Figures 3(b) and 3(c)). Without the disadvantage of T_2_ penetration effect, the center of the lesion presents slightly high signal with low signal in the surrounding of the lesion suggestive of cytotoxic edema, and its periphery suggests vasogenic edema on ADC (Figure 3(a)) [6, 22, 64, 65]. Pathological changes of ANE may explain the above neuroimaging manifestations. Usually, the center of thalamic lesions is perivascular hemorrhage and necrosis of neurons and glial cells corresponding to slightly high signals on ADC, while the periphery of the center portion revealed congestion of arteries, veins, and capillaries and acute swelling of oligodendrocytes corresponding to low signals in the surrounding, with extravasations at the edge of the thalamic lesions corresponding to high signals in the outermost [64]. Taken together, this concentric structure reveals edema, petechial hemorrhage, and necrosis without inflammatory cell infiltration or astrocytic proliferation [12, 21, 25, 32, 35, 59, 69]. It is noteworthy that the typical lesions appear predominantly in the gray matter, especially in the bilateral thalami [2, 64, 65]. The follow-up neuroradiologic examinations of the survivors showed either complete recovery or impressive regression of the lesions, such as atrophy, hemosiderin deposition, and white matter cysts [4, 9, 10, 18–20, 26, 29, 31, 34, 35, 63].

Here we present the neuroimages of an adult patient with ANE who was admitted to our department because of seizure and confusion (Figures 2 and 3). The imaging at onset and during follow-up (two months later) revealed a dynamic change. Hypodensities on CT (Figure 2(a)) and prolonged T_1_- (Figures 2(b) and 2(c)) and T_2_ (Figures 2(d) and 2(e)) relaxation time on MRI of the brain lesions could be found at onset. These lesions were more clear on fluid attenuated inversion recovery (FLAIR) image (Figures 2(f) and 2(g)). At onset, laminar structure but not typical tricolor pattern was found on the bilateral thalami by ADC (Figure 3(b)). Figure 3(a) was a schematic diagram of the typical tricolor pattern of the thalamic lesions (a: center of thalamic lesions characterized by hemorrhage and necrosis; b: periphery of the central thalamic lesions characterized by cytotoxic edema; c: outside portions of the thalamic lesions suggesting vasogenic edema). Besides, we could find brain stem lesions on ADC (Figure 3(c)). While on DWI, these lesions of bilateral thalami and brain stem (Figures 3(d) and 3(e)) appeared without laminar structure. Two months later, these lesions of the brain stem disappeared on T_1_WI (Figure 2(i)), T_2_WI (Figure 2(k)), FLAIR (not shown), ADC (Figure 3(G)), and DWI (Figure 3(I)). Similarly, the lesions on bilateral thalami resolved remarkably, except for some hemosiderin deposition (Figure 2(h): T_1_WI; Figure 2(j): T_2_WI; Figure 2(l): FLAIR; Figure 3(f): ADC; Figure 3(h): DWI; Figures 3(j), 3(k) and 3(l): SWI). Two months later, the patient complained of mild paresthesia around his mouth without any other neurological sequela. The follow-up SWI imaging demonstrated hemosiderin deposition not only in the bilateral thalami, but also in the brain stem and cerebellum.

Gadolinium-contrast MRI has been reported useful in identifying lesions at the very early stage of ANE when conventional CT, MRI, and even DWI and ADC show no abnormalities [37]. This finding suggests that alteration of the BBB permeability might be the first step in the development of brain lesions. The gadolinium-contrast MRI may therefore be helpful for early diagnosis so as to initiate treatment as early as possible and alleviate neurological sequelae of patients [37]. However, Wong and colleagues found that not all ANE patients presented contrast enhancement, partially due to the difference of performing time and the severity of the disease [65]; some unknown pathogenesis of ANE other than the alteration of BBB permeability may also exist [65]. Due to the conflicting results of gadolinium-contrast MRI, further studies are warranted [19, 25, 26, 37].

## 5. Novel Imaging Technologies and Its Clinical Significance to ANE

Some extended or novel imaging modalities have been applied to ANE with clinical significance, which are correlated either to the pathogenesis or to the prognosis of ANE [19, 35, 67, 70]. Moreover, these modalities may contribute to the differential diagnosis of ANE.

Diffusion tensor imaging (DTI) is mainly used for detection of abnormal nerve fibers in the white matter. DTI may potentially picture the microstructural changes in ANE patients with white matter involvement and contribute to the differentiation between ANE and other resembling diseases as well. Chen and colleagues performed DTI in patients with ANE and acute disseminated encephalomyelitis (ADEM) confirmed by biopsy [70]. DTI showed unchanged fractional anisotropy and decreased axial and radial diffusivity suggestive of axonal injury without demyelinationin patients with ANE, which was later confirmed by pathology [70]. However, decreased fractional anisotropy, unchanged axial diffusivity, and markedly increased radial diffusivity compatible with the biopsy finding were found in patients with ADEM, indicative of active inflammatory demyelination [70].

Magnetic resonance spectroscopy (MRS) is another novel modality in neurological clinic. Lipid-lactate complex peak and glutamate/glutamine complex peak on MRS, both of which might be reversible or transient, were found in patients with ANE [6, 26]. The former peak on MRS might be due to the cell membrane damage or disintegration. While glutamate, a well-known excitatory neurotransmitter that may cause neuronal damage, might contribute to the pathogenesis of ANE by glutamate-mediated excitotoxicity if excessive amounts were released into the synaptic cleft. However, the glutamate/glutamine complex peak on MRS was absent from some ANE patients, indicating that the peak might depend on the severity of the disease and may serve as a predictor of outcomes in ANE patients [67].

Additionally, an ANE patient with bilateral symmetrical thalamic lesions received a somatosensory evoked magnetic fields (SEF) examination, suggesting normal latency and strength of the first component elicited by median nerve stimulation [19]. Their findings suggested that thalamus-cortical fibers were intact although thalamus lesions occurred. Their finding was probably ascribed to a reversible course of BBB breakdown and edematous process [19].

In the case of Oki et al., single photon emission computed tomography (SPECT) using ^99m^Tc-ethyl cysteinate dimer (ECD) and* N*-isopropy-*p*-^123^I iodoamphetamine (^123^I-IMP) was performed in an ANE patient on the 39th and 67th days after disease onset [35]. The thalamic lesions presented partially high signal intensity surrounded by low signal intensity on the 34th day after disease onset, indicative of marked hypoperfusion regardless of redistribution on delayed images of ^123^I-IMP SPECT. The irreversible tissue damage despite the recovered blood flow may explain the unfavorable prognosis [35].

## 6. Treatment of ANE

There have been no recommended therapies for ANE thus far. Intensive care, symptomatic treatment and empirical treatment (antiviral therapy), and immunomodulatory agents were tested in a majority of cases [2, 18, 68, 69]. Due to the pathogenesis of ANE, mainly the hypercytokinemia secondary to variable viral infections through immune-mediated mechanism, the immunomodulatory therapy, particularly the therapy that suppressed the cytokine production, has the potential to improve the outcome of ANE. Intravenous glucocorticoids, immunoglobulin, and plasmapheresis should be effective on the basis of the pathogenesis of ANE [5, 20, 24, 25, 30, 57, 60, 63, 68, 69]. Among these therapies, intravenous glucocorticoids, including methylprednisolone and dexamethasone, were the most mentioned and studied, although empirically without systematically determined. However, results from different studies were conflicting. Some researchers reported that administration of steroids within 24 hours after onset or at the early stage of the disease was related to a better prognosis in those without brain stem involvement [13, 18, 56]. However, in spite of the severity of presentation and the late administration of steroids, good outcome was still found in some patients, and some researchers suggested that a trail of steroids should be given to all patients with ANE [10, 17, 20]. Another study, on the contrary, reported that ANE patients treated with steroids had a poor outcome [33]. So far, there has been no consensus on whether we should prescribe steroids to patients with ANE, as well as the dosage, timing, and the duration.

Therapeutic hypothermia, another method of anticytokine therapy, has been used for treating brain swelling caused by trauma and encephalopathy [40, 71]. Therapeutic hypothermia is pivotal to the outcome of the children with ANE, especially if it is initiated within 12 hours after onset [18, 23].

## 7. Prognosis and Its Predictors in Patients with ANE

ANE is a progressive and devastating disease, regardless of treatment. The prognosis of ANE varies from complete recovery to death. It was estimated that the mortality rate was about 30% and less than 10% of patients recovered completely while the neurological sequelae were frequent in survivors [31, 52, 72]. Recently, Bassuk et al. found that the outcome of ANE in Japan was still poor, with incidence of full recovery, mild to moderate sequelae, severe sequelae, and death of 12.8%, 23%, 33.3%, and 28.2%, respectively [13]. Although patients with ANE eventually have a good outcome following a gradual improvement, the course of recovery is slow which results in many children leaving hospital with significant neurologic problems [12, 22, 24, 29, 38, 59]. Hitherto, ANE is still a disabled even fatal disease which should be paid attention to. The reason for the good outcome in some reports may be as follows: (1) increased recognition of the mild form which is diagnosed mainly based on the clinical symptoms, course and radiologic findings; (2) prompt and appropriate treatment after early diagnosis of the disease [3, 18, 19, 21, 23, 31, 60].

Several factors are deemed to be related to the prognosis of ANE acting as predictors. For instance, age above 4 years old was regarded as a predictor of a better outcome while age below 1 year old was indicative a poor prognosis [3]. Moreover, the most mentioned predictive factor in the majority of reports was the laboratory examination, such as the serum aminotransferase and protein in CSF [3], especially the latter, which in the normal range or if was mildly increased usually predicted a better prognosis while moderate or severe elevation was related to a poor outcome [5, 6, 24, 26, 61, 69]. Asymmetric unilateral thalamic involvement and reversion of the image as well were regarded as a predictor of a good outcome while the presence of hemorrhage and localized tissue loss on MRI would predict a poor prognosis [2, 7, 31, 58, 60, 64]. Delirious behaviors, which usually result from brain stem lesions, were considered to be predictive of a poor outcome [57]. The correlation between brain stem lesions and the outcome of ANE was controversial from different reports [12, 31, 32, 38, 44, 45, 63]. However, imaging features seem to be related to the outcome [65]. Wong and colleagues created a scoring system to evaluate patients with ANE [65]. The presence of hemorrhage, cavitation, and location of lesions including the brain stem, the white matter (cerebral, cerebellar, or both) were awarded one point for each of these features, except for the thalami lesions which were 100% involved [65]. They found that the score was related to the outcome of the patients [65]. Another ANE severity scale (ANE-SS) which ranges from 0 to 9 points has been developed, and presence of shock, brain stem lesions, an age over 48 months, a platelet count below 100,000/*μ*L, and an elevated level of CSF protein correspond to 3, 2, 2, 1, and 1 points, respectively [73]. The ANE-SS could be a predictor for the outcome or prognosis of children with ANE [73]. Nevertheless, some extended or novel imaging technologies, as mentioned above, might also be a predictor of the prognosis.

## 8. Diagnostic Criteria for Acute Necrotizing Encephalopathy (ANE)

### 8.1. Diagnostic Criteria for ANE (Proposed by Mizuguchi et al.) [1, 2]

Diagnostic criteria for ANE are as follows:

1. acute encephalopathy preceded by viral febrile disease; rapid deterioration in the level of consciousness, convulsion;
2. increased cerebrospinal (CSF) protein without pleocytosis;
3. neuroradiologic findings for symmetric, multifocal brain lesions involving bilateral thalami, cerebral periventricular white matter, internal capsule, putamen, upper brain stem tegmentum, and cerebellar medulla without involvement of other CNS regions;
4. elevation of serum aminotransferase level to a variable degree without hyperammonemia;
5. exclusion of other resembling diseases:
  - clinical differential diagnosis; toxic shock syndrome, hemolytic uremic syndrome, Reye syndrome, hemorrhagic shock and encephalopathy syndrome, and heat stroke;
  - radiological (or pathological) differential diagnosis; Leigh encephalopathy, glutaric acidemia, methyl malonic aciduria, infantile bilateral strial necrosis, Wernicke encephalopathy, acute disseminated encephalomyelitis, acute hemorrhagic leukoencephalitis, arterial or venous infarct, severe hypoxia or traumatic injury, toxins resulting in symmetric bilateral basal ganglia necrosis (such as carbon monoxide, methanol, 1 methyl-4 phenyl-1,2,3,6 tetrahydropyridine, cyanide, manganese, carbon disulfide, and tegretol), and some other diseases causing symmetric bilateral basal ganglia necrosis (such as osmotic myelinolysis, prolonged hypotension, Canavan disease, methylmalonic acidemia, Wilson disease, Juvenile Huntington disease, striatonigral degeneration, and Hallervorden-Spatz syndrome).

### 8.2. Diagnostic Criteria for ANE1 (Proposed by Neilson et al.) [16, 50]

Except for the above diagnostic criteria for ANE, simultaneously meet any of the following criteria:

1. familial history of neurological symptoms which might be parainfectious;
2. recurrent encephalopathy following fever;
3. additional MRI changes in one of the following: medial temporal lobes, insular cortices, claustrum, external capsule, amygdale, hippocampi, mammillary, and spinal cord.

## 9. Conclusions

ANE is an immune-mediated disease with incompletely recognized pathogenesis. It is underdiagnosed partially due to the insufficient awareness. The diagnosis of ANE is mainly based on the clinical and radiologic features pending the exclusion of other resembling diseases. Immunomodulatory and anticytokine therapies are promising in dealing with ANE whereas more studies are still needed. The prognosis of ANE is variable; however, it is still a potentially devastating disease leading to death and severe neurological sequelae.
